# Barriers to career advancement in the nursing profession: Perceptions of Black nurses in the United States

**DOI:** 10.1111/nuf.12483

**Published:** 2020-07-08

**Authors:** Kechi Iheduru‐Anderson

**Affiliations:** ^1^ School of Rehabilitation and Medical Sciences, The Herbert H. and Grace A. Dow College of Health Professions Central Michigan University Michigan

**Keywords:** Black nurses, career advancement, leadership positions, racial barriers

## Abstract

**Background:**

There is a paucity of Black and minority ethnic group nurse leaders and faculty in the nursing profession, even though the overall number of nurses within this demographic has increased. This study aimed to examine Black nurses' perceptions of the barriers to career advancement in nursing profession in the United States.

**Procedure:**

Participants included 30 Black nurses aged 25 to 65 from health care settings across five US states recruited through purposive sampling. The study used a focused ethnographic design with semi‐structured interviews to elicit responses about participants perceptions and experiences of seeking leadership and faculty positions.

**Results:**

Thematic analysis revealed seven main themes: maintaining white comfort, distrust, no one like me, paving the way, worthy of representation, leadership role not expected of Black nurses, and an advanced degree does not equal advanced opportunities.

**Conclusion:**

The findings suggest that Black nurses face significant challenges in entering leadership or faculty positions. They face racial discrimination and lack access to mentorship and support which discourages sufficiently qualified and experienced nurses from applying for high‐level positions. Ensuring all nurses are afforded equal opportunity for career advancement is essential for the nursing profession's continued growth.

## 1 INTRODUCTION

The overall number of ethnic minority nurses has increased in the United States (US), but the number of Black nurses (BN) leaders and faculty have remained significantly low. There were approximately four million registered nurses in the US in 2017, of which 19.3% identified as an ethnic minority, and only 6.2% identified as Black or African American, less than half their representation in the US population of 13.3%.^1^ The National League for Nursing (NLN) report suggests that nurse ethnic diversity is limited to the frontline nursing staff. Similarly, the NLN nursing faculty census survey of Registered Nurses for 2017 indicated that only 8.8% of nursing faculty are African American compared to 80.8% Caucasian.^2^ The exact number of BN leaders in practice and academic settings have not been specifically reported. However, the Institute for Diversity in Health Management benchmarking national survey of over 6000 US hospitals indicates only 14% of hospital board members, 11% of hospital executive leaders, and 19% of first and mid‐level managers identified as an ethnic minority (2016).

The significantly low number of BNs in senior leadership and faculty positions points to the prevalence of Eurocentric stereotypes in leadership and faculty hiring in the nursing profession.^3^ ^3^, ^4^ report on the future of nursing urged academic leaders to implement measured aimed at the recruitment and retention of nursing students from diverse backgrounds. The report also recommended strategies to increase the number and diversity of nurse faculty, scientists, and researchers. BN leaders are in positions to act and engage with their communities to work towards achieving health equity. With such a significant need to diversify the nursing workforce, we must understand and mitigate issues preventing career progression.

Ethnic minority nurse leaders play an integral role in mentoring, advancing new knowledge, and fostering diverse perspectives among nurses and nursing students who will advance equity in a diverse and global society. However,^5^ expressed great concern for the small number of ethnic minority nurses in leadership, especially in the higher‐level and influential leadership positions. Additionally,^6^ suggested nursing should prioritize preparing racial or ethnic minority individuals to assume greater leadership roles as a means of reducing health disparities. Many colleges and universities have instituted programs and initiatives to increase the diversity of nursing school Bachelor of Science in Nursing (BSN) graduates. However, data from the^7^ indicates that there is a compelling need for colleges and universities “to take a more focused approach to enhance the diversity of the nursing workforce if they are to catch up to, and keep pace with, the country's demographic changes”^8^ (p. 58).

The lack of BNs in leadership and academia is cited as one of the reasons for the poor success of ethnic minority students in nursing, career advancement, and consequently continued health disparity. The AACN recognizes the need to recruit more faculty from the minority population as a strategy for attracting diverse nursing students.^4^ However, the paucity of ethnic minority leaders and faculty undercuts this transfer of political and social capital.^9^, ^10^ Diversity, as stated by,^11^ is a prized resource which should be viewed as a criterion for excellence. Nurse leaders are aware that BNs and other ethnic minority nurses are needed in leadership positions of the nursing workforce to work on policies and procedures affecting Black and other ethnic minority patients. Besides helping students be successful in nursing education, or new nurses acclimate to their new practice environments, good mentors of diverse ethnic backgrounds ensure nurses from similar backgrounds understand that they have a place in the nursing profession and are capable of doing the work.^12^

The experiences of racism are often cited in studies of ethnic minorities in nursing. For instance, a study of managers' perspectives on the promotion of nurses by^13^ indicated that nurse managers felt BNs were not as capable as other nurses and were not motivated to seek higher‐level positions, therefore were denied professional development and promotional opportunities.^14^ identified exclusion and control strategies which faculty of the color stated affected their roles as nurse educators.^15^ reported that African American faculty experienced challenges related to racial discrimination; however, this reinforced their commitment to remain in academia.

As the US racial landscape continues to become more diverse, with the ethnic minority projected to become the majority, it is vital that nursing continues to work diligently to reverse the historical trend of minority group underrepresentation in nursing leadership and academia. It is imperative to understand all factors contributing to the paucity of Black nurses in leadership and faculty roles. A leader in the context of this study is defined as any nurse individual in a position of authority who is responsible for managing staff, patient care, and implementation of the organization's stipulated policies and procedures (nurse managers, directors, educators, supervisors in practice and academia), as well as nurses in academic faculty or instructor roles.

## 2 REVIEW OF LITERATURE

Although cultural diversity and inclusion are routinely articulated as the ethos of the nursing academe and practice, numerous studies examining the experiences of BNs, students, and faculty, as well as other ethnic minority nurses, cite experiences of racism and discrimination.^16^, ^19^ In nursing education, many ethnic minority students experience different types of ethnic and cultural insensitivity at some point during their education^3^, ^20^ which can affect their ability to succeed. However, it has been noted that faculty of color were vital in promoting culture and climate of inclusion for students of color in predominantly white schools of nursing.^21^

There are reports of racism among nursing faculty in the form of exclusionary practices.^14^, ^15^, ^19^, ^21^, ^22^ In a phenomenological study of 15 BN faculty by Whitfield‐Harris et al,^19^ participants reported feeling disrespected, feeling uncomfortable, and students questioning their teaching styles. Some participants reported feeling bullied by students and other white faculty. Many of their participants reported, they felt “discriminated against, discouraged, disrespected, undervalued and unsupported by their white colleagues”(p. 611). Furthermore, BN faculty face significant challenges towards attaining tenure due to limited resources, lack of support, and mentoring as compared to their white counterparts.^14^, ^15^, ^19^

Ethnic minority nurses experienced discrimination, marginalization, and unequal career advancement opportunities.^17^, ^23^, ^24^, ^25^, ^26^ They often felt they had to work harder than their white counterparts to earn the same recognition.^24^ Nurses in^17^ study reported being bullied and treated as less knowledgeable due to minority status and their foreign accent. Many of the nurses left their place of employment due to experiences of discrimination, lack of support, and feelings of exclusion.

Ethnic minority nurses are often made to feel incompetent by their peers through scrutiny, questioning of their work and ability, as well as challenging their work in front of patients and families. A study of 14 Black African nurses by^23^ highlights the everyday experiences of racism faced by these nurses in their workplace. They were stereotyped due to their African origin as lacking the appropriate skills and competency to practice nursing; in addition, they were seen as inferior and lacking in skills required for leadership and management.

Mentoring has been cited as critical to the survival and success of minority nurse faculty^12^, ^18^, ^19^, ^21^, ^27^ and is likened to aspirational barriers.^18^ described an aspirational barrier as what a person feels they can accomplish. Mingo suggests exposure to role models from the same cultural background who are successful in college, as well as meeting faculty members and persons in a leadership role to whom the ethnic minority nurses can relate, can improve their self‐confidence that they too will be able to achieve the same dream. When ethnic minority nurses have mentors who encourage them to reach their potential, it can be a strong motivating factor to “make them feel they possess the skills to accomplish whatever they desire”^18^ (p. 51). This finding is crucial for the development of BNs and students. As Mingo noted, it will take time to increase the number of BN leaders within all nursing programs to be commensurate with the number of Black students, but we need to understand the barrier contributing to the languid pace of increasing the number of BN leaders and educators.

Ethnic minority nurses often described racial discrimination as a barrier to career progression. In a literature review by^28^ exploring the state of knowledge related to minority migrant nurses' experiences of perceived prejudice and discrimination in health care work settings, racial barriers to career progression were commonly described as a form of discrimination. The study also found racial discrimination hindered access to promotion and training with some policies lacking transparency or equitable implementation, “allowing for intentional discriminatory behaviors towards ethnic minority migrant nurses”^28^ (p. 518).

Although racism and racial discrimination have been cited as a barrier in nursing, no study could be found examining the lack of BN leaders as a barrier to the career progression of other BNs. Thus, to the author's knowledge, the extent to which perceptions of racism affects BNs' motivation to seek leadership and faculty positions has not been studied. Additionally, although the lack of BN faculty has been suggested as a barrier to the recruitment, retention, and graduation of Black students, I could not find any studies examining if the lack of BN leaders and faculty is a barrier to BNs' intention to seek leadership or faculty roles.

## 3 PURPOSE

The study aimed to

(1) Examine BN perceptions of the barriers to career advancement in nursing profession in the United States.
(2) Explore whether the dearth of BN leaders and faculty is a barrier to the intents of BN to apply to leadership and faculty positions in nursing.

## 4 METHODS

### 4.1 Design

A focused ethnographic study of 30 BNs was conducted to answer the above research questions using semi‐structured interviews. Focused ethnography is used when a researcher intends to focus on a specific area of inquiry, especially if there are financial and time constraints.^29^ Focused ethnography is appropriate for nurse researchers who plan to “emphasize a distinct issue, situation or ‘problem within a specific context among a small group of people’ living in a bigger society”^30^ (p. 38), and helps the author to understand multiple realities which are socially constructed based on these perceptions. Focused ethnographies may have a limited number of data collection strategies, and participant observation is not a requirement.^31^, ^32^, ^33^, ^34^ Focused ethnography is the method of choice in understanding a more specific aspect of life and cultural experiences relevant to the participants' day‐to‐day experience in their nursing practice.^32^ The facilitation of participants' observation for this study was not feasible due to the diverse employment settings and their geographical location.

### 4.2 Study sample

Thirty BNs aged between 25 and 65 years, with an average of 15 years of experience, and working in different health care settings located within the urban areas in five US states, were recruited using purposive and snowball sampling methods. Twenty‐one of the 30 participants worked in the metropolitan area of the same North Eastern state with a 9% Black population. Purposive sampling ensured the recruitment of participants from the same subculture, who have specific knowledge or experience of interest to the researcher.^32^, ^35^, ^36^ Participants may also recommend other individuals to participate in the study.^30^, ^32^, ^37^ The inclusion criteria were registered nurses (RN) who self‐identified as Black or African American and completion of a BSN with at least 5 years of nursing practice experience.

### 4.3 Ethical considerations

Approval for the study was obtained from the University's Institutional Review Board (IRB). Every participant signed the IRB approved informed consent form before the interview. Each participant was assigned a pseudonym to ensure confidentiality.^35^

### 4.4 Data collection

A short author‐developed survey was used to collect participants' demographic data (Table 2). The 30 individual semi‐structured telephone and skype interviews, of 45 to 60‐minute duration, were digitally audio recorded. The semistructured interview allowed the researcher to ask predetermined questions (Table 1) and delve deeper into the discussion by further exploring additional areas of interest and insights the participants raised during the interview.^38^, ^39^ In addition, it allowed for the discovery of information which is important to participants, but may not have previously been thought of as pertinent by the researcher.^38^, ^40^

**Table 1:** Semi structure interview questions

1.	How would you describe your career experiences as a Black African American nurse in the United States?
2.	How would you describe your career advancement opportunities and experiences?
3.	How have your experience so far affected your motivation to apply to leadership or faculty positions in nursing?
4.	How has the lack of Black nurses in leadership and faculty positions affected your intent to apply for these roles?

### 4.5 Data analysis

The interviews were audio‐recorded, transcribed verbatim, and analyzed using the thematic approach recommended by Burnard et al^41^ and Braun and Clark.^42^ Each audio recording was listened to at least twice to verify the accuracy of the transcription and to make corrections where necessary. The transcripts were read at least two additional times after accuracy was ascertained. This allowed the researcher to become more familiar with the contents, take notes, and add to the spreadsheet used for coding the data. The findings are clustered into themes with supporting verbatim quotes from the participants. Demographic data were analyzed using descriptive statistics (Table 2).

**Table 2:** Participants' demographic data

	Number of participants (N)
Total sample	30
Sex	
Male	4
Female	26
Country of birth	
United States	9
Other Countries	21
Country of basic nursing education	
United States	23
Other Countries	7
Highest level of education	
Bachelor of Science in nursing	13
Master's Degree	12
Doctoral Degree	5
Primary practice settings	
Ambulatory/acute care hospital	20
Long‐term care	5
Academic setting	3
Others	2

Data analysis started immediately after the data was collected from the first participant. When reviewing the audio recording, transcript, and notes taken during data collection, the author identified relevant comments related to perceptions of racism, lack of BN leaders, lack of representation, and how the participant related it to career advancement opportunities. The author carefully grouped and organized these phrases, then checked to determine if they reflected an accurate picture of the participants' story. This was repeated with every participant's transcript and more phrases where added; some were modified, others combined so they represented all the data. During this process, categories were developed which led to the seven main themes representing the study findings.

The analyses was validated through an audit trail, thick descriptions of the study process, as well as reflexivity, to ensure rigor and trustworthiness of the results.^36^, ^43^ The author kept careful notes of all the interviews, including observations made during the interviews, such as changes in the participant tone of voice, prolonged pauses, and change of subject.^35^, ^36^, ^43^ The author engaged in reflexivity by keeping a personal journal to record thoughts, feelings, uncertainties, values, beliefs, and assumptions which emerged during the study.^43^, ^44^, ^45^ This was especially important since the author is a BN who personally experienced racism in both professional and personal life. The transferability of the findings are challenging because “qualitative studies are very unique and the data may not transfer to another study” ^46^ (p. 933).

## 5 FINDINGS

The findings of the study are described under seven themes: maintaining white comfort, distrust, no one like me, paving the way, worthy of representation, leadership role not expected of Black nurses, and an advanced degree does not equal advanced opportunities. Relevant quotes from participants for each theme are presented in Table 3.

**Table 3:** Participant quotes from Interviews organized by the main theme in the order they are discussed in the text

Theme	Example participant quotes			
Maintaining white comfort	I work with nurses who can vouch for my work ethic but will not want me to be their unit manager. Why you may ask, because they are not ready to see a BN, no matter how experienced, or educated, be in charge of them… These white nurses we work with here, they are okay with you working at the same level but not higher. They will prefer a clueless white nurse manager to a smart, opinionated BN.	To your face they will tell you, you are a good nurse, but if it comes to supporting you to advance, they will not support you… As a Black person, you cannot aspire too high in this profession. Doing so disrupts the status quo, it makes many of your white peers uncomfortable, and as a Black person, a BN in America, part of your job is to maintain white comfort.”	I cannot even count how often a white patient has refused care from a BN and immediately the nurse manager and supervisor scrambles to change the assignment, to ensure that the white patient gets a white nurse and is comfortable. But if a Black patient complains about or refused care from a white nurse…that Black patient is labeled difficult, challenging or any other derogatory term you can think of. The Black patient may even be discharged, punished for daring to complain about the white nurse. The Black patient's comfort is not considered, rather the white nurse's comfort is a priority.	In the history of nursing white nurses are leaders, teachers, managers, CNOs, and directors. They control the profession. That's how they want things to be despite evidence to the contrary. Breaking that racial barrier is difficult…. I gave up applying to leadership positions in nursing when I realized that no matter my education and credentials, I was never going to be good enough for them. Two words for you ‘White comfort.”
Distrust	“*They are always watching you closely, looking over your shoulder, like you are doing something wrong. Looking to find a reason to complain about you.”*	“*They don't even trust me to do my job properly as a nurse despite my education and years of experience, they make me feel like I know nothing, and you think that they want me to be a manager?* (laughs). *No. I will not put myself through that, given them more reason to make my life hard.”*	…I am so fearful because I felt like people would be watching my back, watching me closely, people will make sure that I did not succeed, people will not give me the support that I need, to be successful, people will not just trust my judgment or my opinion, even when I have the talent and the competency… So, for me the bottom line for still not applying for a leadership position is fear. I am afraid they will not support me, I am afraid they will be watching me closely, not to help but to find faults, to criticize, to judge. If I thought that I will get the support like my white peers do, I will apply, but I know from experience that I can't trust that I will be supported. I will be judged harshly; my white peers will assume that the reason I got the job is for being Black instead of being qualified and competent to do the job.	I have a master s in nurse leadership, I have worked on this unit now for 15 y, there is a union, and so long as I show up, keep my head down and do my job no one bothers me. I am nearing retirement…But managerial position? I gave up on that a long time ago. It is also an easy way for them to get rid of you. I have seen many BNs come and go quickly because they took a supervisory position. Once you do that, the union cannot protect you. you can get fired for nothing.
No one like me	It was that seeing people of color or Black women in roles of leadership is inspiring. Like Dr. Nora (pseudonym), I don't think you understand. She was my first female Black professor from my country. So, it was inspiring to have her as a professor… I felt like, that's right, so I can succeed in this school and may be like her someday.	I just really admired her so much, and I was like, "Wow! a Black woman can actually get to high levels in nursing!" Especially Black women, like, Dr. Nora, she is a Black woman who has an accent; from a different country. There's a lot of things that you're going to have to deal with. And you were able to overcome all of those things and so she was really inspiring…. when I moved to this state and I saw more BNs in leadership positions, they really inspired me. When there is no one like you doing something, then you think it is out of your reach, that you can't dream that high.	You know racism is just one more obstacle I need to overcome, one more challenge to deal with. Life is full of such things. I love nursing, I love my job as a nurse. there are very few Black It has given me a lively hood many only dream about. I just need to remind myself that I have to work 10 times harder than my white peers to have the same opportunities they have.	
Paving the way	Being challenged and supported by the only BN educator I ever met throughout my nursing education is the reason I ever considered nursing education as a career. She saw me and my potential where the white faculty did not, at least they did not show it. We had a connection that is difficult to explain. She took the time to listen and then challenged me to come back and become an educator if I did not like how things are going for me as a Black nursing student. She stated and I quote, “if you don't like what you see, do something about it.” So, I am here as an educator to do something about it.	I feel like there would be more Black women and men aspiring and applying for leadership positions and higher… having higher career goals if they saw more people that look like them in those positions, so they don't have to feel like, "Oh, I don't belong here." When you see other people like you in those high‐level positions, doing it, you know its within your reach as well if you work hard. You will have more confidence because the main thing like racism and discrimination cannot hold you back, because it did not hold back the other Black person. I think because that other person was able to achieve it, despite the obstacles, then you too can aspire to achieve that as well. It's like that other person has paved the way for you to follow.	I am very ambitious; I like to learn. I asked my Caucasian unit manager to mentor me and give me the opportunity to learn what she does as a unit manager. I cover shifts for her, I volunteer for things other people don't want to do. I told her I can come in when it is convenient for her, even on my days off to work with her, just so I can learn. She said, “why would you want to do that? It is your days off; you should take the time to rest… I get your intentions. Moreover, I don't really have the time to mentor anyone at this time…” I was like, “okay, will you at least think about it? That was 2 y ago.	The facility that I'm at now, where I am a nursing supervisor, when I first started working at the facility as a nurse, my preceptor was a white woman…she said to me "I think you should apply for a job as a per diem supervisor. I think you'll be really good at it…" I was very reluctant to do so. At that time, there was no Black person in management at the hospital. I was the very first BN leader in that place, ever. This was not even 5 y ago. It was the first time that they have ever hired a minority in leadership at any level in that place. They didn't just place me in that position. They supported and mentored me. I felt conformable taking that job and I thrived because I felt accepted and supported.
Worthy of representation	“There were so many things happening at that school before Dr. Joyce (Pseudonym), this Black faculty member came to our program that no one cared about or addressed. We were so afraid to say anything, and when we did no one really cared. But seeing her in that position, as the only Black faculty was empowering, it gave us Black students at the time courage to speak up.”	For once in all the time I was at that institution I felt that I had a voice, that I was being represented. That I will not be dismissed as another Black student who cannot make it and is looking for excuses. This faculty (Dr. Joyce) empowered me. I always prepared well for her class, I wanted her to be proud of me. I felt that she was representing me and people like me, so I needed to do the same for her. It meant a lot to feel represented.	“So, when I first took that class and saw Dr. Kira (pseudonym), I was like blown back, I was like oh my gosh I can't believe they have a Black woman teacher, and she's young, and she's pretty, and she's well educated. I was very glad to see Dr. Kira in that position. I felt connected somehow, I can finally go talk to someone and they can understand what I am saying, really get me, and not just pretending to get me. I felt that finally someone… you know, we are being represented. We are worthy of good representation. These young Black students can dream bigger. Being able to connect to someone who is not a secretary or housekeeper felt really good.	“Let me tell you something, Dr. Kira (Pseudonym) has been an inspiration. A real mentor for all the Black students at that school. We talk about her all the time. She challenged us, made us believe that we can do more than we thought possible. She was kind with her feedback. Did not make people feel stupid. That's important. You can't make people succeed by tearing them down, by destroying their self‐esteem. She expected more, ask for more, but she also gave more. We all had this connection with her…Because of her, I am in graduate school now. She made what I thought was impossible seem possible. She made a difference for a lot of us BNs in that school. We need more people like her to prop us up and support us.”
		So, I worked hard, and I did well in school. It was in large part because of her. The funny thing about her was that she always forced us to look beyond the associate degree, she was always talking about BSN and MSN. I thought she was crazy (laughs). But she was right. When you can see yourself positively through another person, then you know anything is possible.”		
Leadership role not expected of Black nurses	*I think it's just not expected of us to take on a leadership role, being a predominately white profession. They don't expect us to aspire that far*.	…they really want you to stay at the bedside forever. In fact, they prefer that you don't apply for managerial positions, when you do, they don't know what to do with you. Some may even wonder at your audacity to think that you can become a manager. It's like how dare you.	When you are catering to a community of white people, talking to someone who is Black in leadership I think offends them. They don't expect it. I think it's a problem for them, and they don't know how to handle it. So, to protect the white clients from that discomfort of dealing with a Black person in leadership, they will not even consider your application much less hiring you as a nurse manager or supervisor despite your qualifications. If you need leadership experience or position you have to leave. Meanwhile, they hire white nurses with less experience to be managers and supervisors.	*While I was in the North east, despite working in a big hospital in the heart of the city with a master's degree, I couldn't get a job in the specialty units or leadership position. I was denied over and over again. I had to leave the state to get a break*.
An advanced degree does not equal advanced opportunities	You ask for opportunity and you are denied because you do not have the degree. You get the advanced degree, then you ask for the opportunity to learn and grow but you are denied because you do not have any experience. Meanwhile your white peers, even without the education are given opportunities to be managers and clinical educators.		You know it's difficult to break through the racial barriers in nursing to advance. I have decided is not to bother trying anymore. I can count on my one hand how many BN managers are in this big hospital. I work with a lot of BNs with a master's degree who are working as bedside nurses on regular med/surg units. We talk about it all the time. We are not doing this because it is what we want, we just don't have a choice, we've all tried to get advanced positions, transfer to specialty units where our credentials are better suited, but have either been denied, not considered, or blocked altogether. I have watched my white peers with just BSN be offered managerial and other positions that I was denied. My advanced degree means nothing. Nursing is racist, at least where I work. We can try denying it, but it is true.	

### 5.1 Maintaining White comfort

Some of the participants in this study were initially vague at the beginning of the interviews when they talked about the specific details of their experiences. They were reluctant to associate these experiences with racism or racial bias; however, they stated there were no other reasonable explanations for what they experienced or were experiencing. Others were very open and discussed their experiences in great detail, often providing examples of their experiences and comparing them with those of their white counterparts. Some nurses attributed their experiences of racial discrimination to nursing catering to “white comfort.” This experience was described as invading “white spaces,” where the goal is to cater to white people's needs to the detriment of others. Moreover, participants felt some managers' behavior and responses to their plight are geared towards maintaining their status and membership to the in‐group. Others felt that white nurses overall are not ready to have a BN in charge in their workplaces, stating that although their white peers may speak to their abilities and work ethic, many are not ready to be led by a BN because it is not considered the norm.

Some felt having a BN as a unit manager will be disruptive to the way things are. “*You can't disrupt the system*” as stated by one of the participants. Others discussed going through undergraduate and graduate nursing programs without seeing any BN faculty. Furthermore, participants indicated their peers were more comfortable working with them at the bedside but are not willing to support their advancement to higher‐level positions. Another participant related the experience of maintaining white comfort by describing experiences with her nurse manager when white patients refused to be cared for by her as a BN.

*“But if a Black patient complains about or refused care from a white nurse…that Black patient is labeled difficult, challenging or any other derogatory term you can think of. The Black patient may even be discharged, punished for daring to complain about the white nurse. The Black patient's comfort is not considered, rather the white nurse's comfort is a priority.”* [P6] Several participants indicated that the history of nursing as a predominantly white female profession, where most leaders are white, makes it challenging for them to break through the racial barriers. This speaks to a level of distrust expressed by many of the participants for their employers, the system, and the nursing profession. They described the idea of equal opportunity in the nursing profession as an illusion. Several discussed a perceived lack of equal opportunity for all nurses, unequal application of some policies, and disparity in the way some patients are treated within their institutions.

### 5.2 Distrust

Many of the participants discussed a lack of trust in their managers and leaders with some reporting close scrutiny and surveillance by their peers. This was seen as a form of discrimination and a lack of trust in their professional judgment. Two participants stated that they were constantly questioned and interrogated by their peers and managers. It appeared their colleagues and managers did not have faith in their abilities to carry out their nursing duties. For some participants, very close scrutiny and questioning of their competencies was a barrier to seeking leadership positions because it led to a lack of trust. *“They are always watching you closely, looking over your shoulder, like you are doing something wrong. Looking to find a reason to complain about you.” [P4]*

Nurses stated that even if they are qualified for a position, they were unlikely to apply because they felt other nurses would classify them as a token hire. Therefore, while the intent of their recruitment to leadership and faculty position may be to diversify and improve ethnic minority representation, the lack of others like them remains a barrier.

Distrust was expressed by some of the nurses who worked in hospitals with a labor union. They are reluctant to give up the job protection labor unions offered to regular staff nurses and feared the lack of job security accompanying taking on managerial positions.

It is also an easy way for them to get rid of you. I have seen many BNs come and go quickly because they took a supervisory position. Once you do that, the union cannot protect you. you can get fired for nothing.

[P15]

### 5.3 No one like me

Several participants commented that they do not see someone like them in a leadership or faculty position. For some, this meant people who look like them are not expected to be in positions of leadership, while others considered the presence of BN leaders as an inspiration. They felt that they could connect at a different level with them, it also meant they can aspire and succeed in similar roles.

It was that seeing people of color or Black women in roles of leadership is inspiring. Like Dr. Nora (pseudonym), I don't think you understand. She was my first female Black professor from my country. So, it was inspiring to have her as a professor… I felt like, that's right, so I can succeed in this school and may be like her someday.

[P9]

Though some participants reported that not having other “Black nurse leaders and faculty role models makes it hard to get ahead,” they believed that with hard work, advanced education, and continued good evaluations, they will be able to advance in their chosen profession. They viewed their experiences and struggles for career advancement as another obstacle to overcome.

Having more BN leaders and educators are very important for the career development of BNs. Where a BN may attribute negative feedback or poor outcomes to racial prejudice towards him or her, having other Black leaders and faculty within the organization dispels this argument. Mentors can help eliminate the kinds of attitudes by nurses who feel they are not capable of becoming leaders or faculty. They are also uniquely positioned to help their mentees break the racial biases and stereotypes that can suffocate interests and aspirations and help them to realize their full potential.

### 5.4 Paving the way

BN leaders and faculty pave the way for others and understand the challenges associated with completing nursing education and navigating through the rigors of nursing practice. Black mentors can inspire, challenge, and support other BNs. They can help keep Black students in nursing programs until graduation, thus contributing to increased diversity in the nursing workforce.

Many participants are employed in settings where there are few and sometimes no Black counterparts to facilitate career growth, leaving them with limited networks, few mentors, and no meaningful avenues of mutual support. Developing a mentoring relationship exposes nurses of color to jobs and opportunities they may never have heard of or thought they could aspire to, similarly exposing them to people who may never have heard of them.

Some participants reported that they experienced more discrimination when they return to school to advance their degree or when their white peers learned that they are interested in, or applying for, higher‐level positions. Some participants reported asking to be mentored but were rejected by their nurse managers and educators. For some, the rejection was a message that they should not aspire for such positions, while others viewed such rejection as further discouragement from seeking opportunities to learn from their work environment.

Mentoring prepares individuals to become an effective and active participant at any leadership table. Effective mentoring of BNs and other nurses of color bring diversity to the highest levels of decision making in the profession, not just at the lower levels. However, from participant accounts, it appears that finding a willing mentor is a challenge, although not impossible. Mentors for some of these nurses came from nonminority groups as attested by one participant who described how she was offered an opportunity as the first BN in a leadership role at her institution; she was mentored and supported by her white managers, enabling her to succeed and thrive.

Being challenged and supported by the only BN educator I ever met throughout my nursing education is the reason I ever considered nursing education as a career. She saw me and my potential where the white faculty did not, at least they did not show it. We had a connection that is difficult to explain. She took the time to listen and then challenged me to come back and become an educator.

[P13]

### 5.5 Worthy of representation

Having a place at the table is essential to influencing policies that affect the diverse communities served by nurses across the US. BN leaders send the message to BNs that they are worthy of representation at the decision‐making table. They understand the unique challenges facing many other BNs and are in a position to raise and highlight those issues for discussion and consideration when reviewing, revising, and enacting policies. Feeling represented in nursing contributes to the success of Black students and nurses. In particular, BN faculty give Black nursing students a voice and make them feel worthy of representation. Although Black faculty members may not be aware of their influence on students, their presence could contribute to Black and other minority students' success.

There were so many things happening at that school before Dr. Joyce (Pseudonym), this Black faculty member came to our program that no one cared about or addressed. We were so afraid to say anything, and when we did no one really cared. But seeing her in that position, as the only Black faculty was empowering, it gave us Black students at the time courage to speak up.”

[P21]

Some participants' stories included feeling an instant connection with Black educators they had during their education that influenced them. Similarly, several participants spoke about feeling an instant connection to other BN leaders and feeling a sense of belonging which motivated them to aspire for more. This connection to other BN leaders was crucial for these nurses. It showed them that someone cared about them, providing safe spaces to ask questions when they were overwhelmed and allowed for the opportunity to reach out to someone who understood their challenges. This emphasizes how the development of personal and interpersonal relationships is essential for professional development and job satisfaction.

As nursing continues to work to increase minority representation at all levels, providing nurses with mentors of the same cultural background with whom they can relate becomes imperative. Mentors are key to career development and advancement in any field; therefore, BN mentors who are experts, willing and able to facilitate the advancement of BNs' careers, are invaluable assets for these nurses.

### 5.6 Leadership role not expected of BNs

A number of nurses in this study felt that leadership roles were not expected of BNs. Historically, the nursing profession has been dominated by white nurses fueling participants' belief that their white counterparts prefer BNs at the bedside.

…they really want you to stay at the bedside forever. In fact, they prefer that you don't apply for managerial positions, when you do, they don't know what to do with you. Some may even wonder at your audacity to think that you can become a manager. It's like how dare you.

[P17]

The perception that the leadership role is not for BNs was described by many of the participants who worked in community hospitals or hospitals catering to a predominantly white population. One participant discussed working in a community hospital with only five BNs and no person of color in the entire hospital leadership. Another participant, who now lives in the Mid Atlantic area, discussed how her current experience of applying for leadership or specialty unit positions differs from her experience while she practiced in the northeast. Both noted that BNs struggle to advance in many hospitals and are overlooked to employ less experienced white nurses.

### 5.7 Advanced degree does not equal advanced opportunities

Some of the participants reported that their education and credentials were sometimes undervalued and not considered adequate for career advancement. They reported that this was due to nursing being a practice profession and many managerial and faculty positions expect individuals to have supervisory experience; however, “you are not given that opportunity because there is no one like you, who believes in you enough to provide you the experience.” They were assessed as possessing substandard leadership, faculty, and competency skills, thus deeming them unqualified for higher‐level positions away from the bedside. Some workplace policies were often implemented in a way making it difficult for these nurses, despite their educational attainment, to move to higher‐skilled units or leadership positions. Several of the participants believed that earning a graduate degree alone does not ensure career advancement for BNs.

You know it's difficult to break through the racial barriers in nursing to advance. I have decided is not to bother trying anymore…I work with a lot of BNs with a master's degree who are working as bedside nurses on regular med/surg units…We are not doing this because it is what we want, we just don't have a choice…

[P23]

Many of the participants compared their experiences with those of their white counterparts. They indicated their employers offered tuition reimbursement to all employees; although many of them took advantage of it to earn advanced degrees, they were sidelined from advancing to managerial positions. Several believed that because they are not expected to be in these higher‐level positions, they had to leave their places of employment to work in specialty or leadership positions or be forced to remain as bedside nurses. Two participants, in particular, discussed leaving their North East home States to get the opportunity to work in specialty units.

BNs need people in their professional lives who have already learned how to navigate the nuances of the profession. They need collegial, supportive relationships to thrive. Therefore, having more BN leaders who are trustworthy, honest, and willing to support the career development of other BNs is very important. Nurse leaders in every sector need to recognize the value BNs bring to their organizations and institutions, their distinctive knowledge of their communities, and crucial insights they bring with regard to diversity and inclusion in nursing.

## 6 DISCUSSION

The findings of the current study indicate that the lack of BN leaders and faculty influenced the intentions of some of the nurses in the study to seek leadership or faculty positions. Although more than half of the participants had earned a master's degree, many still worked as staff nurses at the bedside, often not by choice. Most participants indicated that given the opportunity and support, they would not hesitate to assume these roles and have applied to such positions previously without success. It is also evident from the findings that these nurses felt they do not have access to the same career advancement opportunities in nursing as their white counterparts. The Sullivan report indicated “that minority nurses place a high value on advancing their education and have a strong interest in moving into upper‐level roles”^47^ (p. 50). The authors of the report expressed concerns at the time that despite the underrepresented minority and BN's remarkable inclination to acquire advanced degrees in nursing, they are significantly underrepresented and less prepared for senior leadership and scholarly roles. Consequently, the commission's report called for the support for “graduate education and leadership development for minority nurses”^47^ (p. 51).

Same race mentoring is essential for professional success. Several participants indicated that they lacked representation in nursing leadership and mentors to pave the way. Indeed, the lack of diverse faculty correlates to the lack of diverse role models to support, mentor, and increase the motivation of diverse and disadvantaged students.^15^ Minority mentors within homogeneous relationships experience greater satisfaction and a sense of contribution than if cultures are mixed. Furthermore, mentors provide mentees with appropriate resources, opportunities, and career options. Unfortunately, few BNs have positions in top management or authority within clinical nursing settings and academia.^5^, ^47^ Compounding the problem is the lack of access to seasoned nurse leaders to formally mentor nurses who seek career advancement to leadership and faculty positions.^48^ Strategic deliberate mentoring works for most nurses seeking new opportunities and experiences.^49^ Specifically, nurses in the current study discussed being inspired to pursue an advanced degree by their BN faculty member(s).

Hassouneh and Lutz ^21^ found that faculty of color were vital in promoting culture and climate of inclusion. Their findings suggested there is a unique nature to the relationship among students of color and faculty of color. This relationship is evident in the results of the current study where participants described what it meant to have BN faculty during their education. BN leaders and faculty play an essential role in mentoring and sharing knowledge about navigating the nursing practice and academic environments. Some nurses in this study reported their relationships with nurse leaders and faculty of the same race and ethnicity were instrumental to their career success. This is consistent with^21^ findings, where participants similarly described these relationships were major contributors to the success of students and faculty of color.

In a study of internationally educated nurses holding management positions,^50^ results indicate that supportive supervisors contributed to the nurses' willingness to accept leadership positions, and the positive working relationships between the participants, peers, and supervisors were instrumental for their success in leadership roles. This finding is similar to the current study with participants who held leadership positions reporting being encouraged and supported by their managers or supervisors and attributed their success to continued support, provision of resources, and opportunities for professional development. Conversely,^51^ indicated that nurses in their study were hesitant to accept managerial roles and declined to participate in governance roles even after receiving encouragement. The current study participants indicated a collective willingness to take on leadership roles but were denied opportunities or were not provided support. Some, however, were unwilling to apply to leadership roles due to prior experiences of lack of support from peers and leaders.

The nurses from this study reported that having graduate degrees did not equal career advancement for BNs. This supports the findings^48^ evaluated a New York University Leadership Institute designed to promote career advancement and leadership in administration, education, and research among nurses of African descent. According to^48^ “more than 80% of the fellows in their two programs held graduate‐level degrees, but only 1 of the 34 Fellows had held a senior management position.” Participants in previous studies also reported that their skills and competencies were devalued, discounting their status as qualified nurses, and leading them to feel untrusted and incapable as qualified professional nurses by their coworkers and some mangers.^19^, ^23^, ^52^, ^53^ ^3^, ^7^ reported some Black overseas nurses in the United Kingdom (UK) reported being placed in nursing home settings instead of their preferred hospital settings, which they found less appealing and with a heavier workload and harder to staff. Many found the experience deskilled them and was not commensurate to their credentials.

Some participants reported experiencing more discrimination when they returned to school to advance their degree or when their white peers learn they are interested in or applying for higher‐level positions. This finding may support some of the participants' speculations that perhaps there is more discrimination when they performed better than their white peers and thus are threatening to them.^54^ reported similar findings of Black Caribbean and African nurses in the UK health care system. These nurses experienced high levels of racial harassment as they advanced in the nursing profession, and presumed that they were viewed as a threat. Discrimination in job promotion and training leads to increased job dissatisfaction and intent to quit. Therefore, as the nursing profession works to increase ethnic minority representation in leadership and academia, all factors affecting job satisfaction and retention must be explored.

This study focused on discovering whether the perceptions of racism impedes BNs motivation to seek leadership or faculty position and if the lack of BN leaders or faculty members are barriers to BNs motivation to seek these positions. The presence of other Black nurse leaders was a source of inspiration to many of the participants. Some of their responses suggest that lack of Black nurse leaders sends a message indicating they should not aspire to higher levels of leadership in nursing. Many in this study believed their racial or ethnic background was a barrier to career progression in nursing. Lack of support and perceived racial biases were overarching themes in this study, indicating leadership support and inclusive practices are crucial to facilitate career advancement for Black nurses. Many of these nurses are very motivated to advance their education, a crucial first step to nursing leadership, which is often denied.

## 7 PRACTICE AND LEADERSHIP IMPLICATIONS

Although there has been an increase in the enrollment and graduation of ethnic minority individuals in nursing, it has not translated into representation in nursing leadership and academia.^2^, ^55^ According to the AACN Enrollment and Graduations data in 2018, a continuous increase in the number of BN graduating with Bachelor of Science, master's, and doctoral degrees in nursing were observed.^7^, ^56^ However, the number of Black or other ethnic minority nurses in executive, mid‐level, or entry‐level leadership or managerial positions was not reported. The lack of consistent and transparent reporting on the number of BNs and other ethnic minority nurses in leadership or faculty positions is concerning for the profession.

Professional socialization (a process of learning the norms, attitudes, behaviors, skills, roles, and values of the profession) is very important for the nursing profession. Lack of BN leaders and faculty conveys a message that BNs should not aspire to these levels in nursing. In contrast, real inclusion in the profession sends the message that these nurses' contributions to the profession are valued. Thus, nurse leaders have significant roles to play in supporting BNs in academia and practice through meaningful inclusion and engagement.^12^

Clearly stated that mentoring could have far‐reaching implications beyond individual career advancement. Indeed, by giving BNs and practitioners the necessary boost in broadening their options and refining their skills, mentoring changes the face of one organization and the makeup of the entire profession. Furthermore, encouraging BNs and other people of color to join, grow, succeed, and stay in the profession expands nursing diversity, one person at a time.

Senior nurse executives are in positions to structure the health care environment, make decisions that direct the flow of resources, and have an enormous influence on reducing and eliminating health disparities that result in quality patient outcomes.^48^ It is important for BNs to have greater participation in healthcare, nursing education, and health policy decision making. There is a need for greater investment in developing and increasing the number of BN leaders and faculty in academia and practice to mentor other BNs at all levels.

Many qualified BNs are prepared to take on leadership and faculty roles with some support from leaders and colleagues. In addition, increasing BN leadership can help reduce the health disparities of minority ethnic populations. To this end, BNs must be actively recruited, developed, supported, and nurtured to assume leadership and faculty roles. BNs understand the health care challenges of their constituents and are in better positions to address the issues that affect the delivery of equitable and culturally appropriate care.

Given the small number of BN leaders and faculty, it is incumbent on nurses of all races and ethnicity to proactively work with students, new nurses, and seasoned nurses within their institutions to reduce the barriers associated with racism which hinder the advancement of the BNs in the profession. Findings from this study indicate that BNs have the skills and academic qualifications to sit at the decision‐making table, are prepared to actively participate in the decision‐making process, and share the perspective of diverse stakeholders they represent.

American Association of Colleges of Nursing^4^ calls for academic leadership and faculty to examine any conscious and unconscious biases that may undermine efforts to enhance diversity, inclusion, and equity, including the use of daily verbal, nonverbal, intentional or non‐ intentional messages which devalue the perspectives, experiences, or feelings of individuals or groups. Experiences of racism and racial discrimination in the profession may hinder the number of Black youths who view nursing as a viable career option. As one of the participants stated, she will never “*advise any youth to become a nurse*” due to her experience of racial discrimination within the profession. To ethnically diversify nursing leadership, we need to recruit ethnically diverse people into the profession. Moreover, by encouraging Black students to take the academic plunge, mentors play a critical role in expanding the nursing universe at the earliest possible juncture.^12^

### 7.1 Limitations

The author's cultural and ethnic background allowed her to form a close and trusting relationship with the participants, which allowed them to speak freely about their perceptions of a sensitive subject. The data presented include the participants' verbatim quotes supporting the identified themes. Since a researcher's cultural and experiential background contain biases, values, and ideologies that may affect the interpretation of a study,^36^ this researcher took steps to ensure the interpretation of the experiences represent those of participants and not of the researcher.^35^, ^36^, ^42^ The author sought to decrease bias by taking the time to interview each participant, ask for clarification of statements, and in some instances, returned to participants to verify their statements. Furthermore, participants may have had a particular motivation to participate in the study, which raises concerns about such influencing factors. Therefore, the findings of this study could only be generalized to the study participants. This is the first study to explore the lack of BNs as a deterring factor for other BNs seeking leadership or faculty positions.

Further research is warranted to explore the factors that contributed to the success of the BNs and other ethnic minority nurses in leadership or faculty roles, and what other resources and development opportunities may be available to assist BN's advancement into these roles. It will be essential to continue to assess the nurses' work environment to develop and test the effectiveness of innovative, evidence‐based strategies aimed at remedying the consequences of unpleasant nursing work environments that have caused these nurses to experience racial prejudice and discrimination, which hindered their career advancement. Findings from such studies will contribute research‐based knowledge to promote further development of conducive work environments where ethnically diverse nurses can thrive and contribute to safe, effective care of diverse patients. The current study did not separate American born Black nurses from Foreign‐born Black nurses, thus a study comparing the perceptions of the two groups would be of interest.

## 8 CONCLUSION

Black nurses benefit from full participation and inclusion in their organizations. Support from leadership and peers is essential for their success. Although many participants in the current study acknowledged the challenges associated with leadership and faculty positions, these challenges were exacerbated by perceived racist behaviors from peers and managers that acted as a deterrent to apply for higher‐level positions. Continued lack of Black nurse leaders and faculty may suggest to potential students that nursing does not value diversity or offer career advancement opportunities. Perceptions of racial inequality in employment and promotional decisions create a hostile and unproductive work environment. Nurse leaders need to examine the career advancement practices within their organizations and ensure that all nurses are afforded equal opportunity for career advancement in the workplace.
